# Recommendations for Choosing the Genotyping Method and Best Practices for Quality Control in Crop Genome-Wide Association Studies

**DOI:** 10.3389/fgene.2020.00447

**Published:** 2020-06-05

**Authors:** Stefano Pavan, Chiara Delvento, Luigi Ricciardi, Concetta Lotti, Elena Ciani, Nunzio D’Agostino

**Affiliations:** ^1^Department of Soil, Plant and Food Science, Section of Genetics and Plant Breeding, University of Bari Aldo Moro, Bari, Italy; ^2^Institute of Biomedical Technologies, National Research Council (CNR), Bari, Italy; ^3^Department of Agricultural, Food and Environmental Sciences, University of Foggia, Foggia, Italy; ^4^Department of Biosciences, Biotechnologies and Biopharmaceutics, University of Bari Aldo Moro, Bari, Italy; ^5^Department of Agricultural Sciences, University of Naples Federico II, Naples, Italy

**Keywords:** crops, GWAS, genotyping, quality control, bioinformatics tools

## Abstract

High-throughput genotyping boosts genome-wide association studies (GWAS) in crop species, leading to the identification of single-nucleotide polymorphisms (SNPs) associated with economically important traits. Choosing a cost-effective genotyping method for crop GWAS requires careful examination of several aspects, namely, the purpose and the scale of the study, crop-specific genomic features, and technical and economic matters associated with each genotyping option. Once genotypic data have been obtained, quality control (QC) procedures must be applied to avoid bias and false signals in genotype–phenotype association tests. QC for human GWAS has been extensively reviewed; however, QC for crop GWAS may require different actions, depending on the GWAS population type. Here, we review most popular genotyping methods based on next-generation sequencing (NGS) and array hybridization, and report observations that should guide the investigator in the choice of the genotyping method for crop GWAS. We provide recommendations to perform QC in crop species, and deliver an overview of bioinformatics tools that can be used to accomplish all needed tasks. Overall, this work aims to provide guidelines to harmonize those procedures leading to SNP datasets ready for crop GWAS.

## Introduction

High-throughput genotyping, which leads to the identification of a large number of single-nucleotide polymorphisms (SNPs) is boosting the implementation of genome-wide association studies (GWAS), linking DNA variants to phenotypes of interest (Taranto et al., 2018). In crop species, GWAS enabled the mapping of genomic loci associated with economically important traits, including yield, resistance to biotic and abiotic stresses, and quality (Boyles et al., 2016; Pavan et al., 2017; Hou et al., 2018; Liu et al., 2018; He et al., 2019). This information has been further used to perform marker-assisted selection (MAS) in breeding programs and discover genes underlying phenotypic variation (Liu and Yan, 2019).

Several genotyping methods are available (reviewed by Scheben et al., 2017), which are usually performed by commercial parties upon the receipt of DNA samples. For application in GWAS, widely adopted genotyping options fall into three categories: whole genome resequencing (WGR), reduced representation sequencing (RRS), and SNP arrays. WGR and RRS are based on next-generation sequencing (NGS) technologies and bioinformatics pipelines that align reads to a reference genome and call both SNPs and genotypes (Nielsen et al., 2011). SNP arrays rely on allele-specific oligonucleotide (ASO) probes (including target SNP loci plus their flanking regions) fixed on a solid support, which are used to interrogate complementary fragments from DNA samples and infer genotypes based on the interpretation of the hybridization signal. Choosing the most appropriate (cost-effective) genotyping method for crop GWAS requires careful examination of several aspects, namely, the purpose and the scale of the study, crop-specific genomic features, and technical and economic matters associated with each genotyping method.

Raw SNP datasets resulting from genotyping experiments are typically inaccurate and incomplete. In addition, genes associated with phenotypes can have a small effect on genetic variance. In this scenario, quality control (QC) procedures are of pivotal importance to minimize false-positive or false-negative associations, referred to as type I and type II errors, respectively. QC includes filtering out poor-quality or suspected artifactual SNP loci, filtering out individuals in relation to missing data, anomalous genotype call and genetic synonymies, and the characterization of ancestral relationships among individuals of the GWAS population. Excellent reviews focused on QC of human SNP data (Turner et al., 2011; Marees et al., 2018); however, the QC procedure may be quite different for crop species. In this case, variables that need to be considered include the crop prevailing mating system (self- or open-pollinating) and the breeding history of the specific GWAS population.

This review aims to provide recommendations on how to plan genotyping experiments and best practices on how to perform QC in crop species.

## Choosing the Correct Genotyping Method

Genotyping methods differ with respect to the number of identifiable SNPs and the cost of the analysis *per* sample, and these two parameters are directly proportional. Given this premise, choosing the correct option for GWAS requires to have a clear idea on two key aspects, i.e., the number of SNPs that is sufficient/desirable to fulfill the GWAS goals and the cost associated with each genotyping alternative. In addition, genotyping methods come with different technical specifications that should be evaluated in relation to the particular GWAS experiment.

### Whole Genome Resequencing

WGR allows the highest number of SNP calls, up to several millions as reported in peach (Cao et al., 2016) and cotton (Du et al., 2018). This is a clear advantage when, rather than MAS, gene isolation is the main aim of the GWAS project (Wang et al., 2016; Happ et al., 2019). Indeed, in high-resolution GWAS, SNP loci showing the highest evidence of association are usually in tight linkage, or may even coincide, with loci underlying phenotypic variation (e.g., Shang et al., 2014; Yano et al., 2016). However, it should be pointed out that, even with a very high marker density, the identification of causal polymorphisms can be difficult in the case of GWAS populations displaying slow decay of linkage disequilibrium (LD) (i.e., populations in which the allelic state at two loci on the same chromosome tends to be correlated even at high physical distance) (Korte and Farlow, 2013). As shown in Table 1, in populations of self-pollinating crops, such as wheat or soybean, the average square correlation coefficient (*r*^2^) between pairs of loci may take several Mb to decay to values indicating substantial linkage equilibrium (0.2 or 0.1) (Vos et al., 2017).

**TABLE 1:** List of some genomic and economic aspects that should be taken into consideration when planning GWAS in crops.

**Species**	**Genome size (Gb)**	**References**	**LD decay**	**References**	**Minimum number of SNPs for a distance < LD decay ^∗^**	**Estimated WGR cost on 100 individuals ($) ^∗∗^**	**SNP array**			
							**Name**	**Technology**	**Size**	**References**
**Brassicaceae**										
*Brassica napus*	0.49	Chalhoub et al., 2014	800 Kb (*r*^2^ = 0.2, A subgenome); 4.8 Mb (*r*^2^ = 0.2, B subgenome)	Zhao et al., 2016	980 (subgenome A) 143 (subgenome B)	19.4 K	International Brassica SNP Consortium	Illumina Infinium BeadChip	52K	Clarke et al., 2016
**Solanaceae**										
*Solanum lycopersicum*	0.90	Sato et al., 2012	665 Kb (*r*^2^ = 0.2)	Ruggieri et al., 2014	1353	36K	SolCAP Tomato 2013	Illumina Infinium BeadChip	9K	Sim et al., 2012
							Axiom Tomato Genotyping Array	Affymetrix Axiom	52K	Unpublished
*Solanum tuberosum*	0.84	Xu et al., 2011	1.5–0.6 Mb (*r*^2^ = 0.1)	Vos et al., 2017	560–14,000	33.6K	SOLCAP Potato 2013	Illumina Infinium BeadChip	10K	Hamilton et al., 2011
							SolSTW array	Affymetrix Axiom	20K	Vos et al., 2015
*Capsicum annuum*	3.30	Kim et al., 2014; Qin et al., 2014	100 Kb (*r*^2^ = 0.2)	Taranto et al., 2016	33,000	132K	UCD TraitGenetics Pepper (Capsicum) Consortium	Illumina Infinium BeadChip	19K	Ashrafi et al., 2012
							Pepper (Capsicum) SNP Genotyping Array	Affymetrix Axiom	640K	Unpublished
**Cucurbitaceae**										
*Cucumis sativus*	0.35	Huang et al., 2009	24 Kb (*r*^2^ = 0.09)	Wang et al., 2018	14,583	14K	–	Fluidigm	35K	Rubinstein et al., 2015
			55–140.5 Kb (*r*^2^ = 0.2)	Qi et al., 2013	6364–2491					
*Cucumis melo*	0.45	Garcia-Mas et al., 2012	100 Kb (*r*^2^ = 0.2)	Gur et al., 2017	4500	18K				
			72–774 Kb (*r*^2^ = 0.2)	Pavan et al., 2017	6250–581					
**Fabaceae**										
*Phaseolus vulgaris*	0.59	Schmutz et al., 2014	1 Mb (*r*^2^ = 0.1)	Diniz et al., 2019	587	23.48K	BARCBean6K_1	Illumina Infinium BeadChip	5K	Song et al., 2015
*Glycine max*	1.12	Schmutz et al., 2010	8.5–15.5 Mb (*r*^2^ = 0.1)	Liu Z. et al., 2017	131–72	44.6K	SoySNP50K	Illumina Infinium BeadChip	6K	Song et al., 2013
			5.9–7 Mb (*r*^2^ = 0.1)	Mamidi et al., 2011	189–159		SoyaSNP180K Axiom	Affymetrix Axiom	180K	Lee et al., 2015
**Apiaceae**										
*Daucus carota*	0.47	Iorizzo et al., 2016	100–400 bp (*r*^2^ = 0.2)	Ellison et al., 2018	4,730,000–1,182,500	18.92K				
**Poaceae**										
*Oryza sativa*	0.39	Sasaki, 2005	150 Kb (*r*^2^ = 0.2)	Liu et al., 2020	2593	15.56K	RiceSNP50	Illumina Infinium BeadChip	50K	Chen et al., 2014
							RICE6K	Illumina Infinium BeadChip	6K	Yu et al., 2014
							Axiom Rice Genotyping Array	Affymetrix Axiom	50K	Singh et al., 2015
*Triticum aestivum*	16.00	International Wheat Genome Sequencing and Consortium, 2014	8 Mb (*r*^2^ = 0.08)	Liu J. et al., 2017	2000	640K	US/Australia 9K Wheat Consortium	Illumina Infinium BeadChip	9K	Cavanagh et al., 2013
							Wheat 90K iSelect	Illumina Infinium BeadChip	90K	Wang et al., 2014
							Axiom Wheat Breeders Genotyping Array	Affymetrix Axiom	35K	Allen et al., 2017
							Axiom Wheat HD Genotyping Arrays	Affymetrix Axiom	817K	Winfield et al., 2016
*Zea mays*	2.50	Schnable et al., 2009	6.34 Kb (*r*^2^ = 0.2)	Dinesh et al., 2016	394,322	100K	MaizeSNP50 BeadChip	Illumina Infinium BeadChip	50K	Ganal et al., 2011
			500 bp (*r*^2^ = 0.2)	Yan et al., 2009	5,000,000		Subset of MaizeSNP50	Illumina Infinium BeadChip	3K	Rousselle et al., 2015
			1.5 Kb (*r*^2^ = 0.1)	Remington et al., 2001	1,666,667		Axiom Maize Genotyping Array	Affymetrix Axiom	600K	Unterseer et al., 2014
							Maize 55K Axiom	Affymetrix Axiom	55K	Xu et al., 2017
**Rosaceae**										
*Malus domestica*	0.74	Velasco et al., 2010	200 bp (*r*^2^ = 0.2)	Larsen et al., 2019	7,420,000	29.68K	RosBREED Apple	Illumina Infinium BeadChip	8K	Chagné et al., 2012
							Fruitbreedomics Apple20k	Illumina Infinium BeadChip	20K	Bianco et al., 2014
							Axiom Apple Genotyping Array	Affymetrix Axiom	480K	Bianco et al., 2016
*Prunus persica*	0.27	Verde et al., 2013	1.2–3.2 Mb (*r*^2^ = 0.1)	Li et al., 2013	221–83	10.6K	RosBREEDPeach	Illumina Infinium BeadChip	9K	Verde et al., 2012
**Vitaceae**										
*Vitis vinifera*	0.48	Jaillon et al., 2007	43 Kb (*r*^2^ = 0.2)	Nicolas et al., 2016	11047	19K	GrapeReSeq Consortium	Illumina Infinium BeadChip	20K	Le Paslier et al., 2013
							GeneChip *Vitis vinifera* (Grape) Genome Array	Applied Biosystems	15K	Unpublished
**Oleaceae**										
*Olea europaea*	1.46	Unver et al., 2017	25 bp (*r*^2^ = 0.05)	D’Agostino et al., 2018	58,400,000	58.4K				
**Malvaceae**										
*Gossypium hirsutum*	2.43	Li et al., 2015	3.2–3.3 Mb (*r*^2^ = 0.1)	Yuan et al., 2018	759–736	97.2K	International Cotton SNP Consortium	Illumina Infinium BeadChip	70K	Hulse-Kemp et al., 2015
			900 Kb (*r*^2^ = 0.1)	Wen et al., 2019	2700		Axiom Cotton Genotyping Array	Affymetrix Axiom	35K	Unpublished

*For several main crop species belonging to different botanical families, the following information is reported: estimated haploid genome size; linkage disequilibrium (LD) decay; the minimum number of equally distributed SNPs providing a distance lower than the LD decay; estimated WGR cost on a panel of 100 individuals; the list of available SNP array(s).*

WGR is especially desirable for GWAS populations displaying rapid LD decay. Indeed, in this case, low marker density may result in missing genomic regions associated with phenotypic traits. Extremely rapid LD decay (in the range of a few base pairs) has been reported for highly heterozygous populations of open-pollinating species (e.g., maize, carrot, olive), in which recombination is effective in breaking up haplotypes (Table 1). In this situation, even in the ideal case of equally spaced SNPs, millions of markers would be required to have a SNP distance lower than the LD decay distance. This is exactly the condition that enables one to detect associations for most genomic regions (Table 1).

WGR genotyping has been so far generally performed using paired-end Illumina technology (e.g., Zhou et al., 2015; Cao et al., 2016; Liang et al., 2019), which, according to our survey, roughly costs $400 per sample for a genome of 1 Gb and 10× average sequencing depth (this term indicating the number of times a base is sequenced on average). This implies that WGR-based GWAS, typically involving a few hundred individuals, may cost several hundred thousand dollars for crops with large genomes, as shown in Table 1. Decreasing the average sequencing depth can lower the cost of WGR; however, this may result in an unacceptable number of genotyping errors. This is especially the case of heterozygous loci, which are associated with a larger number of genotypic combinations (Kishikawa et al., 2019). In practice, WGR in crops has been usually performed with average sequencing depth ranging from ∼5×, as for cotton (Du et al., 2018), tomato (Lin et al., 2014), and peach (Cao et al., 2019), to ∼15×, as for watermelon (Guo et al., 2019) and grapevine (Liang et al., 2019). A notable exception is represented by strict self-pollinating species, such as rice and soybean, for which very low average sequencing depth (1× or lower) has been successfully applied (Wang et al., 2016; Happ et al., 2019). Indeed, homozygous populations of pure lines are effectively haploid, thus allowing easy reconstruction of haplotypes and, consequently, accurate imputation of missing data (Wang et al., 2016).

### Reduced Representation Sequencing

RRS consists in sequencing only a small fraction of the genome, thus reducing the cost of the analysis with respect to WGR (Hirsch et al., 2014). Genotyping by sequencing (GBS) (Elshire et al., 2011), restriction site-associated DNA sequencing (RADseq) (Davey and Blaxter, 2011), and double digest RAD sequencing (ddRAD-seq) (Truong et al., 2012), which use restriction enzymes (REs) for the reduction of genome complexity, are currently the most popular RRS methods used to perform GWAS in crops, mainly due to their moderate cost. At a minimum, this is approximately $35 per sample independently from the genome size and including the application of bioinformatics pipelines for SNP and genotype calling (You et al., 2018). Another advantage of these RRS methods is their scalability, meaning that different combinations of restriction enzymes may be used to customize the percentage of the genome captured.

The number of SNPs identified by RRS genotyping typically varies from a few to several thousands (Pavan et al., 2018, 2019; Colonna et al., 2019), depending on the amount of genome sequenced and population diversity. As discussed above, this output can be largely sufficient in GWAS experiments whose main aim is to implement marker-assisted selection, and for crops displaying slow LD decay (Table 1).

A major technical limitation of RSS is that the genomic distribution of SNPs depends on the specific combination of REs used (D’Agostino and Tripodi, 2017). In addition, sequencing depth at individual SNP loci identified by RRS is typically uneven, leading to under-calling of heterozygous loci and many missing data. The latter issue can be mitigated by genotype imputation strategies; however, we highlight that the success of genotype imputation depends on the genetic makeup of the GWAS population, which influences, among other things, the occurrence of long homozygous segments useful to reconstruct haplotypes (Glaubitz et al., 2014).

### SNP Arrays

SNP arrays for agrigenomics have been developed for over a decade to meet the needs for single research groups or consortia and are still widely used for GWAS in crops despite the decreasing cost of NGS-based technologies (LaFramboise, 2009; Rasheed et al., 2017; Table 1). In 2017, the two leader manufacturers Affymetrix and Illumina had developed 46 SNP array platforms for 25 crop species, associated with a number of markers ranging from 3K to 820K (Rasheed et al., 2017). Pricing of array genotyping is widely considered to exceed that of RRS; however, this is subject to fluctuations over time and is volume-dependent, as it varies with the number of samples and the array SNP density. Indeed, Darrier et al. (2019), considering a set of 1000 barley accessions, found that genotyping with the Illumina 50K iSelect SNP array was cheaper than GBS, with respect to both the cost per sample (£40 vs. £60.50) and the cost per marker (£0.001 vs. £0.003).

From a technical standpoint, SNP array genotyping has a series of advantages. First, genotype calls are generally accurate, even for highly heterozygous species (Bourke et al., 2018). In addition, polyploid crops represent an advantageous field of application of SNP genotyping arrays, as: for allopolyploids, NGS genotyping is complicated by sequence similarity among subgenomes, which hinders the alignment of reads to the reference genome; for autopolyploids, NGS genotyping requires very high sequencing depth and specific polyploid haplotyping algorithms, which make use of the sequence reads to determine the sequence of alleles along the same chromosomes (Motazedi et al., 2018). To date, array providers developed platforms for nine polyploid species ranging from the tetraploid potato to the dodecaploid sugarcane (reviewed by You et al., 2018), together with software solutions suitable to genotype polyploid datasets [i.e., Affymetrix’s Power Tools (APT) and the Polyploid Genotyping Module within Illumina’s GenomeStudio]. We highlight that while GWAS are commonly performed in allopolyploids, GWAS in autopolyploids are complicated by difficulties in the assessment of population structure and allele dosage (Rosyara et al., 2016).

A main disadvantage of SNP arrays is that they suffer from ascertainment bias (Lachance and Tishkoff, 2013), i.e., they cannot identify marker-trait associations in the case of SNPs that were not present in the population used for the development of the array. In addition, a typical drawback in the use of SNP arrays is the possibility that information (e.g., SNP chromosomal location) used for the design of the array is outdated and that there is no consistency in the use of SNPs among different genotyping array formats.

## Recommendations for Quality Control

Genotyping companies apply QC procedures depending on the method used. For NGS genotyping, these consist in removing loci with low sequencing depth (i.e., loci only supported by a few reads) and loci with low PHRED-like quality score (*Q*) (where *Q* indicates the probability that the base call is incorrect). As for array genotyping, these mainly consist in applying a clustering algorithm on fluorescence measurement data of ASO probes to distinguish samples into genotype clusters (allelic discrimination plot), and in assessing a set of QC scores on the goodness of cluster separation and signal-to-background ratio.

**FIGURE 1:**
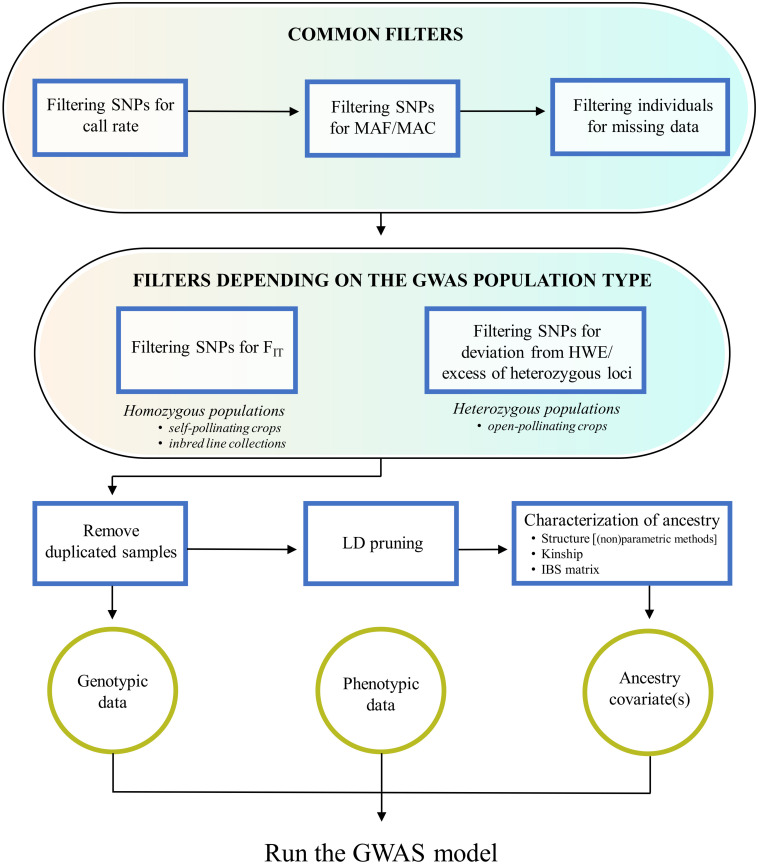
Overview of quality control procedures for crop GWAS. These include: filtering steps that are common to any GWAS experiment; filtering steps depending on the GWAS population structure (homozygous or heterozygous); the removal of duplicated samples; the characterization of ancestral relationships, starting from a SNP dataset pruned for markers in linkage disequilibrium.

It should be clear that, in order to avoid bias and false signals in genotype-trait association tests, the QC procedures above mentioned are not enough and need to be complemented by others performed by the investigator, which are the focus of this paragraph. These include filtering procedures that are either common to any GWAS experiment or depend on the specific GWAS population type, as well as the characterization of the GWAS population for duplicated samples and ancestral relationships (Figure 1).

### Application of Common Filters

**FIGURE 2:**
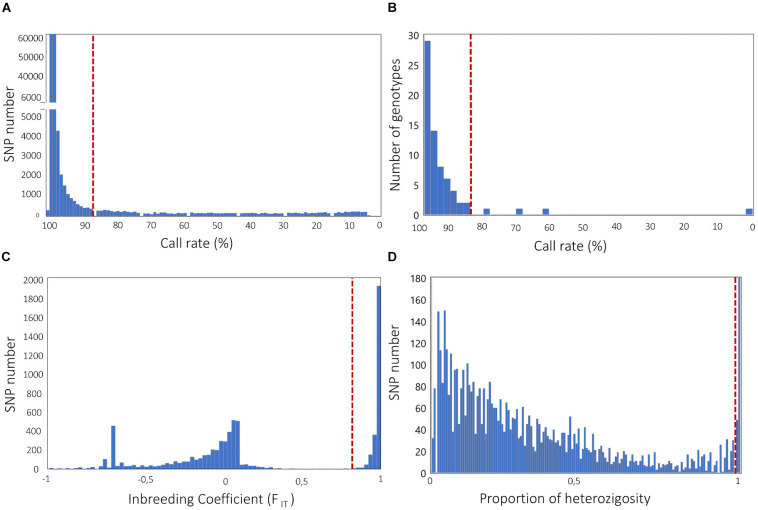
Frequency distribution analysis to define filtering solutions for **(A)** SNP call rate; **(B)** genotype call rate; **(C)** SNP inbreeding coefficient (*F*_IT_); **(D)** SNP proportion of heterozygosity. Dashed lines indicate possible filtering thresholds, based on classes occurring at suspiciously low **(A,B)** or high **(D)** frequency, and distribution gaps **(C)**. Genotypic data used to build histograms are all relative to published genotyping-by-sequencing experiments, carried out in the self-pollinated crops *Cicer arietinum* L. (Pavan et al., 2017, **A,C**) and *Lens culinaris* Medik (Pavan et al., 2019, **B**), and the open-pollinated crop *Cynara cardunculus* L. (Pavan et al., 2018, **D**).

A high rate of missing data at a SNP locus is considered an indication of inaccurate genotype calls (Turner et al., 2011). Therefore, filtering SNPs for call rate is typically the first step in QC. A standard rule is filtering for SNPs with call rates ≥95 or 99% (Anderson et al., 2010); however, a lower threshold might be chosen, especially in the case of NGS genotyping with low sequencing depth. For example, GBS-derived SNP data in crops have been filtered using call rate thresholds of 90% or lower (e.g., Nimmakayala et al., 2014; Pavan et al., 2016, 2017). The overall distribution of call rates may be examined in order to set up a threshold value that eliminates classes occurring at suspiciously low frequency (Figure 2A).

SNP loci displaying rare variants may arise from genotyping errors and, in addition, have low statistical power to reveal association with phenotypic traits, thus they are commonly excluded by QC procedures. In this sense, a widely adopted solution is filtering for minor allele frequency (MAF). Filtering for MAF ≥1–5% has been commonly applied for crop GWAS involving populations of a few hundred individuals (Pavan et al., 2017; Yu et al., 2018), however the same thresholds might be too stringent for larger GWAS populations. Filtering for minor allele count (MAC) allows to set-up thresholds independent from the GWAS population size, commonly ranging from 5 to 10 (e.g., Taranto et al., 2016; Thomson et al., 2017).

As for loci, the presence of individuals with high rates of missing data is also suggestive of technical issues, often related with poor quality and/or quantity of DNA samples. This can generate inaccuracies and bias in downstream analyses. We emphasize that filtering for SNP missingness should normally precede filtering for individual missingness, as the opposite procedure may result in unnecessary removal of individuals. In literature, very different cutoff thresholds for individual missingness have been reported (Begum et al., 2015; Pavan et al., 2018). Our suggestion is to inspect the distribution of missing data across individuals and select a threshold that allows the elimination of classes occurring at suspiciously low frequency (Figure 2B). In addition, for binary traits (e.g., the response to a pathogen, for which individuals of the GWAS population can be classified in either resistant or susceptible), it is of main importance that there are no systematic differences of call rate between the two groups, in order to avoid bias in association tests.

### Application of Filters Depending on the GWAS Population Type

SNP loci characterized by excessive heterozygosity should be filtered out, as they are indicative of technical artifacts or paralogous/repetitive regions that could not be distinguished through the genotyping procedure (Glaubitz et al., 2014). Therefore, specific SNP filters are applied based on the extent of heterozygosity expected in the GWAS population. For crops, this depends on the natural mating system, which may favor self-pollination or open-pollination, and anthropic interventions, such as artificial inbreeding.

Natural populations of self-pollinating crops, as well as populations of inbred lines, are highly homozygous. Therefore, in these cases, even loci with modest heterozygosity rates are suspicious. Glaubitz et al. (2014) suggested the use of the F_IT_ inbreeding coefficient (given by 1-H_o_/H_E_, with H_o_ and H_E_ being the observed heterozygosity and the expected heterozygosity at Hardy–Weinberg equilibrium, respectively) to filter SNPs in homozygous populations, and applied a minimal *F*_IT_ threshold of 0.8 in case of a large population of maize inbred lines. The identification of gaps in the distribution of *F*_IT_ across all loci may help to set up a threshold that allows the elimination of most of the genotyping errors while retaining the highest possible number of loci (Figure 2C).

For natural populations of open-pollinating crops, filtering SNPs that significantly deviate from the Hardy–Weinberg equilibrium (HWE) (e.g., through chi-square or exact tests) can be performed to remove excessively heterozygous loci. In accordance with GWAS on human genotypic data, the HWE filter in open-pollinating crops has been generally applied using a threshold *p*-value of 10^–4^, e.g., in, cassava, olive and watermelon (Anderson et al., 2010; Nimmakayala et al., 2014; D’Agostino et al., 2018; Zhang et al., 2018). We stress here that, in crops, the HWE filter should be used with care, as there is the risk of a significant and unnecessary loss of the GWAS resolution power. Indeed, it should be firstly noticed that the HWE assumption of random mating is not respected when the population has strong genetic structure (see next paragraph) and contains some inbred individuals. Secondly, loci under selection violate by definition the HWE, thus the HWE filter might exclude loci associated with important traits under investigation. All of this considered, solutions might be to (i) adopt a relaxed threshold to eliminate markers, e.g., *p* < 10^–6^, as previously performed on apple and globe artichoke (Bianco et al., 2016; Pavan et al., 2018); (ii) apply the HWE filter separately to each sub-population identified by the analysis of genetic structure; (iii) apply the HWE filter only to individuals not showing the phenotype supposedly under selection, in case of GWAS on binary traits. In other circumstances, including that of partially outbreeding crops, it might be advisable to avoid the HWE filter and, as a possible alternative, to eliminate SNPs with unexpected high levels of heterozygosity (Figure 2D).

### Checking for Sample Duplication and Ancestral Relationships

In the case of crops, GWAS populations might contain several genetically identical samples. This is often caused by the occurrence, in germplasm collections, of unintended duplication of anonymous accessions and/or the occurrence of synonymous accessions. For example, genotyping with the 9K SNP array of the USDA grapevine collection revealed that 568 out of 950 accessions (58%) were genetically identical to at least another accession (Myles et al., 2011).

The identification and removal of duplicated samples is usually performed on the basis of pairwise identity-by-state (IBS) or identity-by-descent (IBD). Pairwise IBS refers to the proportion of alleles shared by two individuals, whereas pairwise IBD refers to the proportion of two individuals’ genome tracing back to the same recent common ancestor (Purcell et al., 2007; Manichaikul et al., 2010). The latter is commonly estimated from pairwise IBS and allele frequency using a method-of-moment algorithm (Purcell et al., 2007). Many studies have used IBS/IBD thresholds of 95 or 99% to declare samples as identical (Myles et al., 2011). The examination of the IBS/IBD distribution associated with a few known identical samples, included on purpose in the GWAS population, might also be used to set up a threshold to estimate identity (Pavan et al., 2019).

Ancestral relationships generate LD between unlinked loci, so they are considered in the GWAS model to limit spurious associations (Astle and Balding, 2009). Therefore, a crucial step in the QC procedure is the characterization of ancestry within the GWAS population. Genetic structure (i.e., the occurrence of sub-populations with different allele frequencies) reflects remote differences in ancestry; in crops, it often originates from physical barriers to random mating and anthropic selection for specific traits, such as seed/fruit size and phenological features (Pavan et al., 2017, 2019; Siol et al., 2017). Instead, kinship reflects recent ancestry, often related to pedigree connections among modern cultivars (Taranto et al., 2020).

Starting from genotypic data, the analysis of population structure can be carried out through different approaches. Parametric methods, such as those implemented in the popular software STRUCTURE (Pritchard et al., 2000) and ADMIXTURE (Alexander et al., 2009), typically estimate the allele frequency of each sub-population jointly with the membership of individuals to each sub-population, using maximum-likelihood or Bayesian statistics. The resulting matrix (known as Q-matrix), which indicates, for each individual, the proportion of the genome referable to various sub-populations, can be conveniently incorporated in GWAS models. However, it should be noticed that parametric methods are based on several genetic assumptions, including those of linkage equilibrium (LE) among markers and HWE within sub-populations. Approximate LE from the original SNP dataset can be obtained by removing markers through LD pruning algorithms (Joiret et al., 2019); on the other hand, HWE may not be met even in populations of open-pollinating crops, due to displacements, breeding activities, and clonal propagation (Campoy et al., 2016).

Non-parametric methods such as principal component analysis (PCA) and multidimensional scaling (MDS) can be used to account for population structure, using coordinates of each individual along the main PCA/MDS axes as covariates in association models (Wang et al., 2009). While non-parametric methods have the advantage of being independent on genetic assumptions, they also come with a number of issues that need to be considered. Importantly, the top PCA/MDS axes may not adequately capture variation due to population structure in the presence of other strong sources of variation, such as outlier sub-populations/individuals or family groups (Price et al., 2010; Liu et al., 2013). These latter may be frequent when the GWAS population contains many cultivars with similar pedigrees. Finally, as for parametric models, it is advisable to perform LD pruning prior to non-parametric analysis, in order to avoid noise from correlated marker data (Liu et al., 2013).

Kinship ultimately depends on the proportion of the genome that is identical-by-descent. Therefore, in order to account for kinship, the GWAS model can use IBD estimates from pedigree notes. However, it is clear that pedigrees of crop species might be in several cases unknown or inaccurate. As mentioned above in this paragraph, methods to estimate pairwise IBD from genotypic data have been also developed. These yield a kinship matrix, also referred to as K-matrix, which has been widely used together with the Q-matrix or PCA/MDS covariates to implement the so-called Q + K and P + K GWAS models (Yu et al., 2006; Zhao et al., 2007).

We finally highlight that several works showed that a simple pairwise IBS matrix could efficiently capture both remote and recent ancestry (Zhao et al., 2007). Therefore, many GWAS models today accommodate the IBS matrix in the framework of linear mixed models, under the assumption that phenotypic variation is positively correlated with genetic distance (e.g., Kang et al., 2008, 2010).

## Bioinformatics Tools to Perform QC

QC can be carried out using several bioinformatics tools, which may differ with respect to the specific action(s) performed and the file requested as input. Therefore, the investigator is often called to the conversion of genotypic data among different formats, the most common being variant call format (VCF), haplotype map (hapmap), pedigree/map (ped/map), binary (bed/bim/fam), Affymetrix chip (chp), Illumina sample map and final report, and structure. PGDSpider^1^ (Lischer and Excoffier, 2012) is a dedicated tool for the conversion of genotypic data among a wide range of formats. Among other powerful conversion tools, we mention the one implemented in the software suite TASSEL (Bradbury et al., 2007), which deals with the most common formats associated with NGS genotyping, and the *gene_converter* function within the R package radiator (Gosselin, 2017), accepting and delivering 13 and 29 file formats, respectively.

Several open-source software suites are available for QC. Among the most widely used, PLINK (Purcell et al., 2007), starting from common genotypic data file formats (ped/map, bed/bim/fam and VCF), enables the application of all the SNP and individual filters presented in Sections “Application of Common Filters” and “Application of Filters Depending on the GWAS Population Type,” with the exception of the *F*_IT_ filter. In relation to the study of genetic ancestry, it has options for LD pruning and MDS, and for the estimation of pairwise IBS and IBD.

Compared with PLINK, the abovementioned TASSEL (Bradbury et al., 2007) accepts a wider range of file formats (also including hapmap) and does not perform filtering for HWE departure. On the other hand, having been developed for GWAS on maize inbred lines, TASSEL provides the possibility to perform the *F*_IT_ filter. As for the genetic ancestry options, it can perform PCA/MDS and estimate pairwise IBS. While PLINK is based on command lines, thus requiring specific training by the user, TASSEL also implements a graphical user interface. Another important feature of TASSEL is the possibility to easily build histograms for SNP and individual missingness and SNP heterozygosity, which, as discussed above, are useful to set up cutoff thresholds specific for each GWAS experiment.

Investigators with some bioinformatics skills may be interested in QC tools also enabling filtering procedures depending on the genotyping method, which, as stated above, are commonly performed through external services. For NGS genotyping, we cite VCFtools (Danecek et al., 2011), a command line software suite developed for the VCF format, which allows, among other options, filtering SNP sites and individuals based on sequencing depth and PHRED-quality score. For array genotyping, we cite the following: (i) the proprietary packages GenomeStudio and Axiom Analysis Suite, for data generated on Illumina or Affymetrix SNP array platforms, respectively; (ii) freeware tools that directly accept raw data in the original format generated by array genotyping platforms, including fluorescence intensity data necessary for QC of genotype calls. Among the many available options, we cite here the R packages argyle (Morgan, 2016) and SNPQC (Gondro et al., 2014), and the Python package ASSIsT (Di Guardo et al., 2015), for data generated on Illumina SNP array platforms, and AffyPipe (Nicolazzi et al., 2014), for data generated on Affymetrix SNP array platforms.

Finally, concerning the study of genetic structure, besides the above mentioned STRUCTURE (Pritchard et al., 2000) and ADMIXTURE (Alexander et al., 2009), the EIGENSOFT utilities SMARTPCA and SMARTEIGENSTRAT are popular bioinformatics tools for, respectively, detecting and analyzing population structure via PCA, and correcting for population stratification in association studies (Price et al., 2006).

## Conclusion

This work is thought to provide researchers, who mainly focus on the biology and breeding of crop species, with essential technical and economic aspects required to plan and carry out cost-effective and accurate GWAS. To the best of our knowledge, this is the first work specifically addressing the issue of QC in crop species, so we expect that it may contribute to the future harmonization of the procedures leading to the obtainment of high-quality SNP datasets ready for GWAS.

## Author Contributions

SP, ND’A, and EC conceived the review. SP, ND’A, EC, and CD wrote the manuscript. CL and LR critically revised the manuscript.

## Conflict of Interest

The authors declare that the research was conducted in the absence of any commercial or financial relationships that could be construed as a potential conflict of interest.

## Footnotes

1 http://www.cmpg.unibe.ch/software/PGDSpider/
